# Comment on: Effects of changes in trunk inclination on ventilatory efficiency in ARDS patients: quasi‑experimental study

**DOI:** 10.1186/s40635-023-00565-9

**Published:** 2023-12-11

**Authors:** Francesco Marrazzo, Stefano Spina, Francesco Zadek, Roberto Fumagalli, Thomas Langer

**Affiliations:** 1 Department of Anesthesia and Critical Care, ASST Grande Ospedale Metropolitano Niguarda, Milan, Italy; 2 Department of Medicine and Surgery, University of Milan-Bicocca, Monza, Italy

**To the editor:**

We have read with great interest the study conducted by Benites et al., which explores the impact of trunk inclination on ventilator efficiency in patients with ARDS [1]. Similarly to our own studies [2–4], and understandably given the historical period, most studied patients (18/22) had COVID-related ARDS. However, some results presented by Benites differ from what we have described and hypothesized [2–4] and might, therefore, deserve further discussion.

The first key issue is the potential influence of trunk inclination on PEEP titration. Our recent work revealed that the “best PEEP”, balancing overdistension and collapse (identified with electrical impedance tomography-EIT), varies by an average of 5 cmH_2_O, depending on trunk inclination (semi-recumbent at 40° vs. supine-flat), with lower values identified in semi-recumbent position [4].

Although the authors do not specify the trunk inclination of patients during PEEP titration, their observations of significant improvements in respiratory mechanics and CO_2_ clearance in the supine-flat position suggest that PEEP may have been optimized in this position. In our opinion, it is highly plausible that such improvements would not have occurred if PEEP had been optimized in the semi-recumbent position. Indeed, in our recent study we observed similar lung compliance in supine-flat and semi-recumbent position, once PEEP was titrated considering trunk inclination [4].

Another methodological issue that needs to be discussed is the use and comparison of absolute end-expiratory lung impedance (EELI) values. While changes in EELI within the same position are a useful proxy of lung aeration, caution is advised when interpreting absolute values and their differences according to trunk inclination. Indeed, these changes might be influenced by altered pressure on the electrodes and/or minor belt movements induced by the postural change itself.

Beyond these methodological aspects, the major discrepancy with our findings is ventilation distribution. In a similar study conducted in volume-controlled ventilation, thus ensuring a constant tidal volume, we observed improved ventilation of the ventral area in supine-flat position [3]. Given the anatomical location, the rapidity of improvement, and the quick reversibility, we attributed the improved respiratory mechanics mainly to a reduction of ventral alveolar hyperinflation and a specular increase in regional compliance. In contrast, Benites and colleagues hypothesize that the mechanism underlying the improved respiratory function in the supine-flat position is enhanced dorsal ventilation, and thus dorsal regional compliance. Indeed, they describe a reduction in ventral ventilation in favor of the dorsal distribution of tidal volume, indicated by a reduction in impedance ratio (calculated as the ventral tidal impedance change divided by the corresponding dorsal impedance change). Notably, the tidal volume-induced impedance changes reported by the authors do not exhibit significant variations with trunk inclination.

In conclusion, we think that some technical and methodological aspects require careful consideration when interpreting the findings. In addition, and in consideration of our current understanding of respiratory mechanics and physiological gravitational effects on aeration and ventilation distribution [5], it would be important to hypothesize potential and plausible mechanisms explaining the reduced ventral and increased dorsal ventilation observed in the study.

## Data Availability

Not applicable.
